# Testosterone and Adult Neurogenesis

**DOI:** 10.3390/biom10020225

**Published:** 2020-02-03

**Authors:** Mark D. Spritzer, Ethan A. Roy

**Affiliations:** 1 Department of Biology, Middlebury College, Middlebury, VT 05753, USA; 2 Graduate School of Education, Stanford University, Stanford, CA 94305, USA; ethanroy@stanford.edu

**Keywords:** testosterone, adult neurogenesis, androgen, hippocampus, dentate gyrus, sub-ventricular zone, BDNF, neuroprotection, spatial memory

## Abstract

It is now well established that neurogenesis occurs throughout adulthood in select brain regions, but the functional significance of adult neurogenesis remains unclear. There is considerable evidence that steroid hormones modulate various stages of adult neurogenesis, and this review provides a focused summary of the effects of testosterone on adult neurogenesis. Initial evidence came from field studies with birds and wild rodent populations. Subsequent experiments with laboratory rodents have tested the effects of testosterone and its steroid metabolites upon adult neurogenesis, as well as the functional consequences of induced changes in neurogenesis. These experiments have provided clear evidence that testosterone increases adult neurogenesis within the dentate gyrus region of the hippocampus through an androgen-dependent pathway. Most evidence indicates that androgens selectively enhance the survival of newly generated neurons, while having little effect on cell proliferation. Whether this is a result of androgens acting directly on receptors of new neurons remains unclear, and indirect routes involving brain-derived neurotrophic factor (BDNF) and glucocorticoids may be involved. In vitro experiments suggest that testosterone has broad-ranging neuroprotective effects, which will be briefly reviewed. A better understanding of the effects of testosterone upon adult neurogenesis could shed light on neurological diseases that show sex differences.

## 1. Introduction

Determining the neural mechanisms by which new memories are formed is a fundamental question in the field of neurobiology, and the study of adult neurogenesis has provided exciting new insights that directly address this question [1,2]. Adult neurogenesis involves the proliferation, migration, and differentiation of new neurons within the adult brain. Although there is some evidence that adult neurogenesis occurs in other brain regions [3,4], neuronal stem cell populations have been well characterized in only the subgranular zone of the dentate gyrus region of the hippocampus and the subventricular zone (SVZ) of the mammalian brain [5]. Cells produced in the SVZ migrate along the rostral migratory stream to differentiate into functional interneurons within the olfactory bulbs [6]. Most of these new neurons integrate with the main olfactory bulb, which is responsible for processing and responding to olfactory cues detected by the main olfactory epithelium, while some new neurons also become part of the accessory olfactory bulb, which processes pheromonal cues detected by the vomeronasal organ [6]. Newly proliferated neurons from the subgranular zone of the dentate gyrus migrate a short distance into the granule cell layer of the dentate gyrus, where they extend functional axons into the CA3 region of the hippocampus [7,8,9]. Young hippocampal neurons exhibit enhanced excitability, increased Ca^2+^ conductance, and a lower threshold for induction of long-term potentiation (LTP) than do mature granule cells [10,11,12], which may make them a particularly good substrate for memory formation. Additionally, increased adult neurogenesis shortens the persistence of LTP within the hippocampal circuit, which facilitates the formation of new circuits and associated memories [13].

Adult neurogenesis occurs in most mammalian species studied to date [14,15], but the amount of neurogenesis declines with increasing age [15] and, because of this, the functional significance of adult neurogenesis in humans remains controversial [16]. Neurogenesis was first documented in post-mortem subjects that had been injected with 5-bromo-2’-deoxyuridine (BrdU) to label the progression of tumors [17]. The BrdU was incorporated by many cells in the dentate gyrus, with approximately 22% of the cells co-expressing neural markers indicative of neurogenesis. Another study used levels of ^14^C in post-mortem hippocampal genomic DNA derived from nuclear bomb testing to estimate that adult humans add 700 new neurons to the hippocampus every day, corresponding with 0.004% of the dentate gyrus per day [18]. Other studies using endogenous markers of cell proliferation and neurogenesis suggest that neurogenesis rates decline dramatically after four years of age in humans, becoming nearly undetectable by adolescence [19,20]. However, this result has been countered by more recent work showing robust expression of cell proliferation and neurogenesis markers well into old age (i.e., individuals in their 90s) [21,22,23]. The controversy seems to stem from different methods of measuring sub-populations of proliferating cells, and substantial evidence now indicates that markers of neurogenesis (e.g., doublecortin and PSA-NCAM) are expressed in the hippocampus of adult humans.

Adult neurogenesis is a multi-stage process [24,25], and the steroid hormone testosterone could influence any stage of development. Two components of adult neurogenesis are routinely measured: the number of newly proliferated cells produced, and the number of cells that survive to specific time points. Dividing cells can be marked by injecting subjects with thymidine analogues that are incorporated into the replicating DNA, and BrdU has been the most commonly used analogue [26]. BrdU-labeling allows cells to be precisely birth dated based on the timing of injections and brain tissue collection, and co-labeling with neuronal and glial markers allows quantification of cellular differentiation. A variety of endogenous proteins that are transiently expressed during different stages of neural development can also be used to assess cell proliferation and neurogenesis [24,27]. Two of the more commonly used markers are Ki67 and doublecortin (DCX), which are expressed in proliferating cells and early developing neurons, respectively [28,29,30,31].

Adult neurogenesis may be a mechanistic link that allows steroid hormones to influence cognitive ability. Numerous past studies have shown that sex steroids influence adult neurogenesis both in the SVZ and the dentate gyrus, and a number of recent reviews have been written on this topic [32,33,34]. This review will focus more narrowly on the effects of testosterone upon adult neurogenesis and cognition. The idea that testosterone could influence adult neurogenesis stemmed initially from observed sex differences in levels of cell proliferation and cell survival within the adult brain [35]. Although this review will focus on the mammalian condition, some of the first findings of sex differences in neurogenesis came from studies with birds [36], which will be briefly reviewed. Studies with rodents have shown either higher levels of cell survival in the dentate gyrus in males than females [37,38,39] or no sex difference in cell survival [40,41,42,43,44]. The effect of sex on cell proliferation has also been inconsistent, with studies with rodents showing no sex difference [42,43,45], higher levels in females [40,46], or higher levels in males [44,47]. Part of this inconsistency may be due to cyclic variation in estrogens among females: Tanapat et al. (1999) demonstrated that female rats have greater levels of cell proliferation than males only during proestrus, when circulating estradiol levels peak. Despite inconsistencies, initial findings suggesting sex differences in neurogenesis have led to productive research demonstrating that testosterone plays an important role in regulating adult neurogenesis.

## 2. Testosterone Biosynthesis and Metabolism

**Figure 1:**
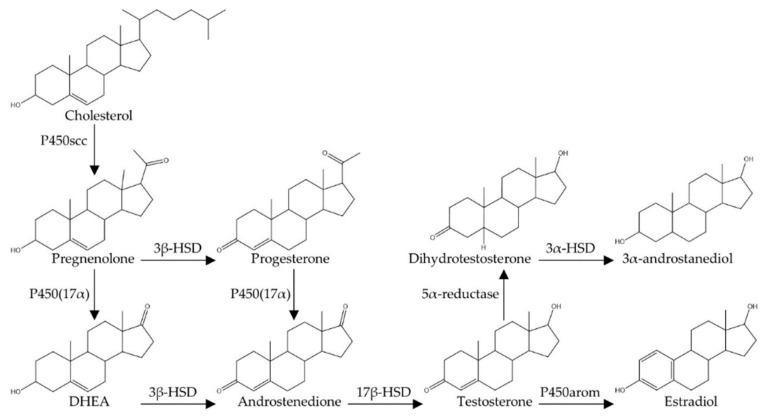
Key sex steroid synthesis pathways found in the gonads and the brain. Enzyme name abbreviations are as follows: P450 side-chain cleavage enzyme (P455scc), 17α-hydroxylase [P450(17α)], 3β-hydroxysteroid dehydrogenase (3β-HSD), 17β-hydroxysteroid dehydrogenase (17β-HSD), 3α-hydroxysteroid dehydrogenase (3α-HSD), and P450 aromatase (P450arom) [48,52,54].

Testosterone is an androgen, meaning that it is a 19-carbon steroidal derivative of cholesterol (Figure 1) [48]. Typical of steroid synthesis, cholesterol is first converted to pregnenolone within mitochondria by the side-chain cleavage enzyme (P450_scc_). Subsequently, pregnenolone is converted to a variety of different androgens [e.g., dehydroepiandrosterone (DHEA) and androstenedione] by other P450 enzymes, which are heme-containing proteins involved in electron transport chains along the membrane of the endoplasmic reticulum [48,49]. In male mammals, including humans, testosterone is the dominant circulating androgen. In men, 95% of circulating testosterone (6–7 mg/day) is produced by the Leydig cells of the testes [49]. Although testosterone production by Leydig cells is 7 to 8 times higher than that produced by the ovaries in females, circulating testosterone also has significant effects on female physiology [50]. It is also noteworthy that all of the key enzymes for testosterone production have been localized in the rat and human hippocampus, indicating that some testosterone is produced de novo within the brain itself to act as a neurosteroid [51,52,53].

The hypothalamus-pituitary-gonadal axis (HPG axis) plays an essential role in regulating early development, adolescence, and sustaining the adult reproductive functions. In this hormonal axis, the hypothalamus secretes gonadotropin-releasing hormone (GnRH), which then stimulates the release of the gonadotropins, luteinizing hormone (LH) and follicle-stimulating hormone (FSH), from the anterior pituitary into the blood stream [49]. LH plays a critical role in regulating the production and release of testosterone by the testes. Additionally, the HPG axis is regulated by a negative feedback loop in which GnRH neurons are hyperpolarized by estradiol [55]. This feedback loop prevents excess release of gonadotropins and reduces levels of circulating androgens and estrogens.

In the brain, testosterone either binds directly to androgen receptors (though its affinity for these receptors is relatively low) or is broken down into either dihydrotestosterone (DHT) or estradiol. The enzyme 5α-reductase breaks down testosterone into DHT, and 5α-reductase has been localized to many neural structures, including the hippocampus and cerebral cortex [56]. DHT has been shown to have an approximately two-fold higher binding affinity to androgen receptors and a five-fold slower dissociation constant from these receptors compared to testosterone [57]. Testosterone is also converted into estradiol in the brain through P450 aromatase [58], and this enzyme has been localized to all regions of the hippocampus, including the dentate gyrus [59]. Estradiol acts through two known intracellular receptors (ERα and ERβ) to have genomic effects as well as through a G-protein coupled receptor (GPER) to have more rapid non-genomic effects [60]. Furthermore, a metabolite of DHT, 3α-androstanediol (5α-androstan-3α,17β-diol), can bind to neuronal ERβ to have genomic effects [61], and there is evidence that 3α-androstanediol can enhance memory in male rats through an estrogen-receptor dependent pathway [62,63]. Therefore, although DHT cannot be aromatized to estradiol, it remains possible that it is acting on estrogen receptors via conversion to 3α-diol.

## 3. Testosterone and Neurogenesis in the Avian Brain

The study of the effects of testosterone upon adult neurogenesis was pioneered by researchers studying seasonal changes in the song-control nuclei of birds. The high vocal center (HVC) is a component of the song system used for the production and learning of song, and in the adult brain new neurons migrate into this region from the walls of the lateral ventricles in a manner comparable to adult neurogenesis within the olfactory bulbs of rodents [36]. Male canaries show a seasonal peak in neuron number within the HVC that corresponds with song learning during the breeding season, and these changes seem to be driven by seasonal changes in testosterone [36]. Specifically, Alvarez-Buylla and Kim (1997) noted that pyknosis within the HVC is highest during periods of seasonal decline in testosterone, and they suggested that low testosterone induces cell death, providing vacancies for newly proliferated cells to grow into. Testosterone implants given to female canaries caused an increase in neurogenesis within the HVC via enhanced cell survival but not cell proliferation [64]. However, a subsequent study demonstrated that testosterone implants given to both female and male canaries caused an increase in cell proliferation specifically along the ventral portion of the ventricular zone [65]. Similarly, male starlings (*Sturnus vulgaris*) given testosterone implants showed an increase in HVC volume and an increase in the number of actively proliferating cells along the ventricular zone [66].

The effects of testosterone upon neurogenesis within the avian HVC seem to involve both androgen and estrogen pathways, as neither estradiol treatment alone nor DHT treatment alone enhanced neurogenesis within the HVC of female canaries, while a combined treatment with DHT and estradiol enhanced neurogenesis to levels comparable to testosterone treatment alone [67]. Additional experiments with canaries demonstrated that testosterone-induced neurogenesis within the HVC of female canaries was proceeded by enhanced capillary vasculature (angiogenesis) and the production of growth factors [68,69].

Birds also display seasonal changes in adult neurogenesis within the hippocampus [70,71]. For example, free-ranging black-caped chickadees (*Parus articapillus*) show a peak in hippocampal neurogenesis during the fall, when they are also engaging in the most caching behavior (a spatially demanding behavior) [72]. However, there is no sex difference in hippocampus size or caching behavior in chickadees [73] and so it seems unlikely that sex steroids regulate seasonal changes in hippocampal neurogenesis in this species. Brown-headed cowbirds (*Molothrus ater*) are a brood parasite, with females engaging in the spatially demanding task of laying their eggs in the nests of multiple hosts during the breeding season. Female cowbirds show greater hippocampal neurogenesis than males, and neurogenesis levels peaked after the breeding season was over [74]. The researchers suggested that this seasonal change facilitated the replacement of old neurons with new ones during a period (non-breeding season) when there was less demand upon hippocampal memory [74]. They also found that testosterone levels were lower in both males and female cowbirds outside the breeding season, suggesting that testosterone might suppress hippocampal neurogenesis in cowbirds, but this idea has not been tested experimentally.

## 4. Testosterone and Adult Neurogenesis in the Olfactory Bulbs

Compared to the dentate gyrus, relatively little work has been done testing the effects of testosterone upon neurogenesis in the mammalian olfactory bulbs (Table 1) [75]. Studies with mice and rats have shown that females have higher levels of cell proliferation within the SVZ than do males [76,77,78,79]. Estradiol treatment had no effect on cell proliferation in the SVZ of female mice [80], suggesting that it is not the cause of the sex difference in cell proliferation. Similarly, exposure to either testosterone or estradiol had no effect on cell proliferation of mouse SVZ cells grown in culture [77]. For both female rats and mice, prolonged exposure (14–21 days) to estradiol caused a decrease in neurogenesis within portions of the olfactory bulbs [80,81], although the relative effects on the accessory and main olfactory bulbs differed between the two species. A study with adult male mice demonstrated that castration increased cell proliferation within the SVZ [78], suggesting that testosterone suppresses cell proliferation in this region. In contrast, castration caused a decrease in cell proliferation in the SVZ of juvenile male rats, and proliferation levels were restored by testosterone or estradiol injections but not by DHT injections [82], which suggests that testosterone is acting through an estrogen-dependent pathway. It is unclear whether the difference between the two studies is due to the species used or the developmental stage tested (the mice were tested at 6-8 months old, whereas the rats were only one month old). Assuming no species difference, one would conclude that testosterone enhances cell proliferation in the SVZ of males via an estrogen-dependent pathway prior to adolescence, while in the SVZ of adult males testosterone suppresses cell proliferation. More work needs to be done to determine whether the effects testosterone on cell proliferation in the SVZ lead to changes in neurogenesis within the olfactory bulbs.

Considerable evidence indicates that newly generated neurons in the olfactory bulbs play a critical role in odor discrimination broadly and pheromonal signaling among adults in particular [6,83]. As one relevant example, transgenic mice (Sema7A knockout) that have reduced GnRH release from the hypothalamus, and therefore reduced testosterone levels from birth, showed no preference for female odors over male odors in adulthood (i.e., they lacked typical male sex preferences) [84]. These transgenic animals showed a significant increase in neurogenesis within the accessory olfactory bulbs in response to exposure to male urine [84], a response not observed in wild type males but typical of wild type female mice. Sixteen days of testosterone treatment given to adult transgenic mice reversed this effect. Castration had a behavioral effect similar to that caused by the Sem7A knockout (i.e., castrated males preferred investigating male odors over female odors), and testosterone injections in adulthood reversed this effect. Importantly, testosterone injections suppressed neurogenesis in the olfactory bulbs of males exposed to male odors [84], indicating that testosterone plays an important regulatory role in neurogenesis within the olfactory bulb that facilitates sex preferences among adult male mice. This is a dramatic example of a hormone changing perception and, in turn, changing behavior.

## 5. Testosterone and Adult Neurogenesis in the Dentate Gyrus

### 5.1. Testosterone and Stages of Neuronal Development

Some of the earliest work showing that testosterone influences adult neurogenesis in rodents involved testing seasonal changes in neurogenesis among meadow voles (*Microtus pennsylvanicus*). Male meadow voles show seasonal changes in testosterone, with a peak during the breeding season [85], and males with higher testosterone levels have a larger hippocampus [86]. These seasonal differences in hippocampal volume may be a result of testosterone’s effect on new cell survival within the dentate gyrus. In support of this hypothesis, reproductively active male meadow voles (with high testosterone levels) had greater levels of new neuron survival compared to reproductively inactive male voles [87]. In contrast, reproductively active and reproductively inactive males did not differ in the amount of cell proliferation occurring within the dentate gyrus [85,87], suggesting that seasonal fluctuations in androgens may enhance cell survival but not cell proliferation. However, a more recent field study demonstrated that cell proliferation and neurogenesis decline in both male and female voles during the breeding season relative to the non-breeding season [41]. Unlike prior work, the voles in this field study spent no time in captivity. Given that no sex differences in neurogenesis were observed, it is reasonable to conclude that variables other than sex steroids (e.g., stress hormones, age, or diet) can have an over-riding influence on seasonal changes in neurogenesis in wild rodent populations [41].

Experiments with laboratory rodents support the general conclusion from the early studies with voles; namely, that testosterone enhances adult neurogenesis in the dentate gyrus by increasing cell survival, while having little or no effect on cell proliferation (Table 2). Among adult male rats, castration had no effect on cell proliferation within the dentate gyrus but caused a significant decrease in the survival of new neurons, as measured 24–30 days after BrdU injection [88,89]. When castration was conducted prior to puberty (30-day-old rats), rather than in adulthood, there was still no effect upon hippocampal cell proliferation [90]. Similarly, castration did not cause a reduction in Ki67-expressing cells in the dentate gyrus of male mice [91] or rats [92], supporting the general conclusion that testosterone does not play a significant role in regulating cell proliferation. Numerous experiments, involving a wide range of testosterone doses, have also demonstrated that testosterone replacement or supplementation have no effect on cell proliferation in the dentate gyrus of castrated or intact adult male rodents [93,94,95,96,97,98]. There are, however, two reports showing that castration caused a decrease hippocampal cell proliferation in adult male rats [89,99], but this effect seems to be subtle given that most studies demonstrate no effects on castration or testosterone administration on cell proliferation.

Concerning cell survival effects, 30 days of testosterone replacement (injections or implants) significantly increased neurogenesis in the dentate gyrus compared to castrated control rats [88,100]. Similarly, 35 days of testosterone exposure via slow-release pellets enhanced neurogenesis levels among intact male mice compared to non-testosterone-treated intact males [96]. Interestingly, shorter periods (15–21 days) of testosterone replacement did not increase neurogenesis relative to castrated control rats [93,94,101]. Spritzer et al. (2011) found that castrated rats had significantly lower levels of neurogenesis than intact controls when a 16-day cell survival period was used, but 15 days of testosterone injections did not reverse the effects of castration. The dose of testosterone used (0.500 mg/rat) was a relatively high physiological dose, suggesting that lower physiological levels of testosterone, as observed in intact rats, may enhance neurogenesis while higher doses do not. Similarly, high doses of the testosterone analogue 19-nortestosterone caused a significant decrease in 5-day cell survival in the dentate gyrus [102]. However, 30 days of a high physiological dose of testosterone (0.500 mg/rat) enhanced neurogenesis [88], which would suggest that different doses of testosterone influence the different stages of neurogenesis in different ways. There is also evidence that low doses of testosterone have no effect on the survival of new neurons, based on both 30 days of injections (0.125 mg/rat) [88] and testosterone implants that produced a low dose for 26 days [103]. In summary, a relatively high physiological dose of testosterone given over a prolonged period (approximately 30 days) enhances neurogenesis within the dentate gyrus of male rodents by increasing cell survival.

Thus, current evidence indicates that the later stages of neural development are sensitive to the neurogenesis-enhancing effects testosterone, while the effects of testosterone on cell proliferation and early stages of neurogenesis seem to be minimal. It is unclear whether 24 (or more) consecutive days of testosterone exposure is needed to enhance adult neurogenesis, or if acute bursts of testosterone during specific stages of neuronal development would have the same neurogenesis-enhancing effect. A supra-physiological dose of testosterone given to male mice during early cellular development (0–2 days after birth) had no effect on subsequent survival of 28-day-old cells [104]. Castration was found to reduce the number of DCX-labeled cells among male mice and rats [91,99]. DCX is expressed during the cellular migration of synaptic integration stages of development [110], suggesting that testosterone may influence this critical period. In support of this idea, an unpublished experiment from our laboratory demonstrated that five days of testosterone injections given during synaptic integration (11–15 days old) increased new cell survival, while injections given earlier in development (1–5 and 6–10 days old) had no effect on cell survival [111]. As the dendritic arbor grows during the later stages of development, the newly developed neurons receive excitatory glutamatergic input from the entorhinal cortex and send glutamatergic signals to the CA3 region [112], and so testosterone may influence neurite extension in some way. The period during which testosterone seems to influence neurogenesis (11–30 days after birth) corresponds roughly with the period during which GABAergic input from neighboring cells within the dentate gyrus transitions from being excitatory to inhibitory [113,114], and GABAergic signaling has been shown to promote neuronal maturation [115,116]. The testosterone metabolite 3α-androstanediol has been shown to be a positive allosteric modulator of GABA_A_ receptors [117], and so perhaps 3α-androstanediol enhances GABAergic signaling onto newly generated neurons to, in turn, enhance their survival. Interestingly, 4–6 weeks of age has been identified as a critical period in neuronal development, when new neurons exhibit enhanced long-term potentiation and are preferentially recruited into neural networks for use in spatial memory tasks [12,118]. Furthermore, environmental enrichment increased network connectivity for new hippocampal neurons among mice that were exposed to the enrichment during a critical period when new neurons were 2–6 weeks old [119]. This leads to the exciting possibility that short-term testosterone exposure could enhance neural plasticity during a critical time window and, in turn, facilitate memory formation. As previously mentioned, most work to date on testosterone and neurogenesis has involved testing either cell proliferation or cell survival broadly, but future work attempting to pinpoint the specific stages of neural development that are influenced by testosterone will need to take into account the fact that new neurons mature approximately twice as fast in rats compared to mice [110].

### 5.2. Metabolites of Testosterone

Testosterone can be aromatized to estradiol or converted to DHT in the brain (Figure 1) [49,120], suggesting that the effects of testosterone on neurogenesis may be through an androgen-dependent or an estrogen-dependent pathway. Experiments with rats and mice that have tested the relative importance of DHT and estradiol in regulating neurogenesis in males suggest that testosterone’s effects on adult neurogenesis are via an androgen-dependent pathway. Thirty days of DHT injections given to castrated male rats increased neurogenesis in a manner similar to testosterone, whereas 30 days of estradiol injections had no effect on neurogenesis [88,100,105]. Fifteen days of estradiol injections also had no effect on hippocampal cell proliferation or survival among male rats, while causing an increase in cell proliferation and a decrease in new cell survival among age-matched female rats [43]. Further supporting an essential role for androgen receptors, testosterone implants did not increase neurogenesis (30-day-old cells) in castrated male rats that had non-functional androgen receptors due to a testicular feminization mutation (TFM), and co-treatment of castrated male rats with DHT and an androgen receptor antagonist (flutamide) blocked the neurogenesis enhancing effects caused by 30 days of DHT injections alone [100]. Similarly, flutamide was shown to block an exercise-induced increase in hippocampal neurogenesis (2-week-old neurons) among male rats, whereas an estrogen-receptor antagonist (tamoxifen) did not [92]. Prolonged DHT injections (37 days) increased hippocampal neurogenesis in male mice, but somewhat unexpectedly this effect was not observed in mice that were induced to selectively over-express androgen receptors in the brain [106]. The authors speculate that excessive androgen receptors in newly developing neurons may reduce neuronal survival in some way, comparable to some neurogenesis-impairing effects that have been observed with high doses of testosterone [101,102]. Paralleling the findings for testosterone replacement, DHT injections had no effect on levels of cell proliferation in the dentate gyrus of rats, mice, or voles [97,100,105,106]. However, blocking DHT production using finasteride caused a decrease in both cell proliferation and DCX-expressing cells within the hippocampus of male mice [107]. This effect of acute DHT depletion did not persist, however, as there was no difference in the number of new neurons 35 days after the last finasteride injection [107]. This supports similar findings obtained with acute injections of testosterone [104]. It is unclear why blocking DHT production decreased cell proliferation and systemic DHT injections had no effect on cell proliferation, but this could indicate that blocking endogenous androgen production throughout the body (including in the brain) [121] influences cell proliferation in ways that manipulating only circulating androgen levels does not.

Testosterone could be influencing hippocampal neurogenesis in adult males via its metabolite, estradiol, but relatively few experiments have tested this idea. During five-day injection periods during different stages of neural development, estradiol injections increased neurogenesis in the dentate gyrus of adult male meadow voles only during the axon extension phase of neural development; namely, days 6–10 after cell birth and not during earlier (1–5 days) or later (11–15 days) periods of development [108]. Fifteen and 30 consecutive days of estradiol injections had no effect on neurogenesis in male rats [43,88], suggesting a species difference or a differential effect of prolonged exposure to estradiol compared an acute (5 day) burst of estradiol during the cell migration period. Middle-aged male mice (10–12 months old) given estradiol implants showed an increase in cell proliferation and new neuron production (DCX-expressing cells) but no change in 21-day cell survival [109]. This result contradicts findings that an acute dose of estradiol has no effect on cell proliferation among mice or voles [97,104,108], and this discrepancy may be due to either an age effect (young vs. middle-aged) or duration of dosing. Thus, there is some evidence that acute doses of estradiol increase cell proliferation and possibly early neuronal development, but these effects do not correspond with the timing of the neurogenesis-enhancing effects of prolonged testosterone exposure. It is therefore unlikely that the effects of testosterone upon neurogenesis in male rodents involve an estrogen-dependent pathway.

There is, however, considerable evidence that estradiol regulates hippocampal neurogenesis in females [32], demonstrating a clear sex difference in the regulation of adult neurogenesis by sex steroids. Acute removal of estrogens reduced cell proliferation in female rats [40], whereas long-term ovariectomy has no effect on cell proliferation in female rats or mice [42,122]. Acute injections of estradiol enhanced cell proliferation in female rats [122,123,124,125], while cell survival of new neurons can be enhanced or suppressed by chronic estradiol exposure in females contingent on the timing of estradiol replacement [43,126,127]. To our knowledge, only one study to date has investigated the effects of androgens upon hippocampal neurogenesis in females. A 30-day treatment with DHT that enhanced neurogenesis in male rats had no effect on neurogenesis in young or middle-aged female rats [105]. Thus, current evidence points to an apparent sex different in the role of sex steroids in regulating hippocampal neurogenesis, with an androgen-dependent pathway playing a dominant regulatory role in males and an estrogen-dependent pathway playing a dominant role in females.

### 5.3. Testosterone, Neurogenesis, and Spatial Memory

It has long been known that the hippocampus plays a critical role in spatial memory formation [128,129,130], and so it is not surprising that most work testing the function of hippocampal neurogenesis has involved testing rodents in spatial memory tasks [131]. Spatial memory refers to encoding, storing, and recalling spatial information about surroundings, positions of objects, or specific routes [132]. Stress impairs both adult neurogenesis and performance on various spatial memory tasks among rodents [133]. Similarly, decreased performance on spatial memory tasks correlates with decreased adult neurogenesis among aged rodents [8,134,135]. On the other hand, environmental enrichment increases hippocampal neurogenesis and improves performance on hippocampus-dependent tasks [136,137,138]. Additionally, a number of studies have shown that performance on a variety of spatial memory tasks results in increased adult neurogenesis. For example, rats that engaged in the hippocampus-dependent Morris water maze had more neurogenesis in the dentate gyrus than did rats engaging in a cued version of the water maze that is hippocampus independent [139,140,141]. Furthermore, the specific neurons that were promoted to survive by training in the water maze, were selectively activated when mice were re-trained in the same maze [142]. Blocking hippocampal neurogenesis using chemicals or irradiation resulted in decreased short-term and long-term memory retention in the water maze [143,144]. Addiontally, genetic techniques used to block hippocampal neurogenesis in transgenic mice were shown to impair memory acquisition and retention on the water maze [145], and similar results were obtained using a lentiviral technique to reduce neurogenesis in rats [146]. Besides influencing spatial memory broadly, some elegant experiments have demonstrated that hippocampal neurogenesis is specifically involved in pattern separation [147] and in preventing proactive interference during spatial tasks [148].

The effects of sex steroids upon the brain are commonly divided into early developmental effects (organizational) and effects that occur in the adult brain (activational). Experiments with humans and rats have shown that testosterone has organizational effects upon the brain that enhance spatial learning and memory [149,150]. More relevant to the relationship between testosterone adult neurogenesis, there is also considerable evidence that testosterone has activational effects that differentially influence the working and reference components of spatial memory. Working memory is a form of short-term memory that involves storage of relevant information while completing a specific task, whereas reference memory involves long-term storage of information from one task to be used for the next task [151]. Using a rat model, we have demonstrated that testosterone enhances spatial working memory in a dose-dependent manner but has no effect on spatial reference memory [152,153], which corroborates previous findings [154,155,156,157]. However, experiments using the object location memory task (OLMT) suggest that testosterone also influences some forms of long-term memory. Specifically, testosterone replacement was shown to improve spatial memory among castrated young adult male rats on the OLMT following both a 30 min retention period [158] and a 2 h retention period [152,159]. Thus, testosterone enhances spatial working memory and some forms of spatial reference memory, but a critical question is whether these memory improvements are the result of changes in adult neurogenesis. This remains largely untested, but it is noteworthy that neurogenesis is critical for acquisition of the reference memory version of the water maze [143,145,160] and testosterone injections can improve memory on this task [156,161,162]. Considerably more evidence indicates that testosterone improves spatial working memory on radial-arm maze tasks [152,154,155,156,157,163,164], and there are some studies showing that hippocampal neurogenesis plays a role in this type of maze task [147].

A handful of experiments have more directly addressed the hypothesis that testosterone improves spatial memory by increasing adult neurogenesis in the dentate gyrus. One study examined the relationship between sex differences in neurogenesis among rats and their performance on a trace-eye-blink conditioning task, which is hippocampus dependent [38]. Without training on the task, males had higher levels of neurogenesis (12-day neuron survival) than did females, but after training the females had higher levels of neurogenesis than the males [38]. This difference after training corresponded with better performance by females on the task, suggesting that a sex difference in hippocampal neurogenesis leads to a sex difference in cognitive ability. Whether or not this difference is the result of activational effects of steroid hormones remains unknown. A more relevant study compared performance by male and female rats on a spatial pattern separation task and measured neurogenesis in the same animals [165]. It was found that males that used a spatial strategy to solve the more challenging version of the task (i.e., distinguishing between adjacent maze arms) also had higher levels of neurogenesis (27-day cell survival) within the dorsal hippocampus than did females trying to solve the same task. This sex difference in neurogenesis corresponded with better performance by males than females on this task [165], but again the role of sex steroids in regulating these sex differences is unclear. Shin et al. (2016) tested the effects of castration upon hippocampal neurogenesis and spatial memory in the same animals. They demonstrated that castration caused a decrease in 3-day cell survival and number of DCX-labeled cells, while also causing a decrease in spatial working memory on a radial-arm maze task [99]. Clearly, more work is needed in this area, particularly testing whether the observed effects of testosterone on later-stage neuronal survival have any impact on spatial memory.

## 6. Possible Molecular Pathways within the Hippocampus

Because steroids are hydrophobic, androgens pass directly through cell membranes to bind to cytoplasmic androgen receptors that dimerize and act as a transcription factors [166], and the resulting changes in gene transcription could explain the effects of androgens on neurogenesis. A comparison of the genes expressed within the hippocampus of intact and castrated male rats revealed 98 genes that were upregulated and 173 genes that were downregulated by castration [167]. The implications of these wide-ranging changes have not been well characterized, but it is noteworthy that NMDA receptors and GABA receptors were up-regulated in the hippocampus by castration [167], possibly compensating for reduced connectivity. Using a more focused approach, another study assessed changes in mRNA expression for specific genes involved in synaptic plasticity within the hippocampus of males rats within 9 days of castration [168]. Castration caused a decrease in transcripts for brain-derived neurotrophic factor (BDNF) and synaptophysin, while increasing expression of transcripts for acetylcholine receptors (α7-nicotinic and M_1_ muscarinic) and an NMDA receptor subunit (GluN1). The authors suggest that the upregulation of receptors may have been a compensatory response to reduced synaptic signaling caused by down-regulation of synaptophysin and BDNF [168].

Androgens could bind directly to receptors on cells within the adult hippocampus to influence their survival directly. Androgen receptors and androgen receptor mRNAs have been localized within the CA1 sub-region of the hippocampus of adult male rats [169,170]. Although at lower levels than in the CA1, androgen receptor mRNA and protein expression has also been detected in the dentate gyrus of male rats [102,162,171] and mice [172]. Androgen receptors were also found in neural stem cell cultures developed from cells obtained from the subventricular zone of adult female rats [102]. Therefore, androgens could increase hippocampal neurogenesis by binding to androgen receptors on newly proliferated cells. However, there is contradictory evidence suggesting that androgen receptors do not occur in the dentate gyrus of male rats [100,169,170,173]. These contradictory findings may be due to various methodological differences, with the age of the rats being one critical variable: middle-aged male rats had significantly more ARs in the dentate gyrus than did young male rats [105]. Another important factor seems to be the strain of rats used, as high levels of AR expression were found in the dentate gyrus of Wistar rats [102,162], low levels were observed in Sprague-Dawley rats [105,171], and no AR expression was observed in the dentate gyrus of Fischer 344 [169] and Long-Evans rats [170]. Additionally, ARs were not observed on young DCX-expressing neurons in the dentate gyrus of young adult male mice and rats [100,106], which led Hamson et al. (2013) to propose that androgens act upon the CA3 pyramidal cells, which have abundant AR expression, to indirectly influence neurogenesis in the dentate gyrus. This hypothesis remains to be tested.

Testosterone, or its metabolites, may influence adult neurogenesis by altering levels of neurotrophic factors within the brain [174]. For example, female canaries given testosterone implants showed increased levels of BDNF, and BDNF infusions increased neuron survival within the HVC [175]. Louissaint et al. (2002) provided evidence indicating that testosterone enhances production of vascular endothelial growth factor (VEGF) within the HVC, while also increasing the number of VEGF receptors on local capillary beds [68]. The resultant increase in angiogenesis led to elevated production of BDNF by endothelial cells, which may, in turn, enhance neurogenesis within the HVC. Some evidence suggests that similar interactions between testosterone and trophic factors influence neurogenesis within the rodent hippocampus. Both BDNF and VEGF have been associated with increased survival of newly proliferated neurons within the dentate gyrus [176,177,178]. Male rats have higher levels of BDNF within the dentate gyrus than do females [179], suggesting that BDNF levels may be regulated by sex hormones. In support of this, castration decreased BDNF levels in the hippocampus of male rats [99,168,180], and testosterone implants increased hippocampal BDNF in a transgenic male mouse model of Alzheimer’s disease (SAMP8) [181]. Surprisingly, neither DHT nor testosterone influenced BDNF levels within the hippocampi of aged male rats [153,154]. The lack of an effect of DHT specifically, may be because testosterone is influencing BDNF after being converted to estradiol. An estrogen-response element has been well characterized within the *Bdnf* gene [182], whereas androgen receptor binding sites within the *Bdnf* gene remain speculative [183]. Thus, BDNF may be upregulated in the male hippocampus by estradiol, as has been documented for female rodents [184,185], but this would not explain the evidence that testosterone acts via an androgen-dependent pathway to enhance neuronal survival in the hippocampus. Additionally, the effects of testosterone upon BDNF levels have been found to vary among the sub-regions of the hippocampus. Specifically, castration increased BDNF levels within the mossy fibers extending from the dentate gyrus to the CA3 layer of the hippocampus [186,187], which directly contradicts the hypothesis that testosterone is up-regulating BDNF in the dentate gyrus to, in turn, enhance adult neurogenesis. However, castration decreased BDNF production within CA1 layer of the hippocampus [188], which also has high levels of androgen receptors, suggesting that testosterone may enhance neurogenesis through indirect mechanisms involving enhanced CA1 plasticity.

Androgens have been shown to have a wide range of neuroprotective effects, which may or may not involve activation of neurotrophic factors [189]. The idea that testosterone is influencing neurogenesis via increased neuroprotection fits well with findings that testosterone enhances neurogenesis mainly through increased neuronal survival rather than through changes in cell proliferation. Androgen treatment protects against neuronal damage caused by kainate lesions [190], oxidative stress [191], ischemia [192] hyperphosphorylation of τ protein [193], and β-amyloid toxicity [190,194,195,196,197]. Although some of the neuroprotective properties of testosterone may occur through aromatization to estradiol [198], evidence indicates that neuroprotection also occurs via androgen-dependent pathways [199,200]. For example, DHT implants caused a significant reduction in neuronal loss in the hippocampus following kainite lesions among castrated male rats [190]. The neuroprotective properties of androgens involve activation of the mitogen-activated protein kinase (MAPK) pathway [201,202], and the MAPK pathway has been shown to induce increased neuron survival in a wide range of contexts [203,204]. Specifically, Gatson et al. (2006) found that DHT induced phosphorylation of MAPK through a genomic pathway and enhanced the phosphatide 3-kinase pathway through membrane-bound receptors. Rat hippocampal cells in culture were used to show that testosterone and DHT had neuroprotective effects specifically against toxins that activated apoptosis, but these androgens were not protective against toxins that acted through non-apoptotic pathways [205]. Androgens may, therefore, activate the MAPK pathway to have anti-apoptotic effects [206], which, in turn, increase the survival of newly developing neurons in the adult brain. In support of this hypothesis, castration of male rats was found to increase levels of multiple protein markers of apoptosis, including caspase-3 [99]. Additionally, testosterone implants given to castrated male rats were found to up-regulate MAPK1 (also called ERK2) within the hippocampus, and this was associated with reduced depressive-like behavior [94]. Androgens also rapidly phosphorylate CREB in cultured hippocampal neurons, which can have neuroprotective effects, but this effect was found to involve the protein kinase C (PKC) pathway rather than the MAPK pathway [207]. Therefore, there is evidence that multiple intracellular pathways are activated by androgens to enhance neuron survival.

## 7. Interactions between HPG and HPA Axes

Some complex interactions occur between the hypothalamic-pituitary-gonadal axis (HPG axis) and the hypothalamic-pituitary-adrenal axis (HPA), which regulates the stress response. For example, castrated male rats show elevated release of corticosterone in response to restraint stress compared to intact males exposed to the same stressor [208]. This occurs because testosterone inhibits arginine vasopressin synthesis in the hypothalamus, which reduces basal adrenocorticotropic hormone (ACTH) levels, which, in turn, maintains relatively low corticosterone levels [209]. A variety of chronic stressors impair spatial working and reference memory in rodents [210,211,212], and the negative effects of stress on spatial memory seem to be caused by corticosterone binding to receptors in the hippocampus [213,214]. These negative effects of stress upon spatial memory can be modulated by testosterone. For example, testosterone replacement in castrated rats reduced anxiety and enhanced cognitive performance on an inhibitory avoidance task [215]. Furthermore, a male advantage in a water maze task was eliminated when rats were given preliminary trials to reduce stress [216,217]. Similarly, eliminating the hormonal stress response via adrenalectomy also eliminated sex differences in water maze performance [216]. Assuming a positive relationship between adult neurogenesis and spatial cognition, these results suggest that the neurogenesis-enhancing effects of testosterone may be partially explained by its neuroprotective effects against glucocorticoids.

In general, acute and chronic stress cause a decrease in adult neurogenesis [133], and this effect is likely mediated through elevated glucocorticoid levels caused by stress [218,219]. Sex differences have been observed in the neurogenic response to various stressors [37,220], suggesting a possible regulatory role for testosterone. Among male rats, physiological levels of testosterone prevented the neurogenesis-reducing effects of social isolation, whereas high doses (0.500 mg/rat) provided no such buffer [101]. Castration caused an increase in depressive-like behaviors among male rats in response to chronic mild stress, and this was associated with decreased cell proliferation and neurogenesis (25 day survival) relative to sham-castrated control rats [89]. Another study with male rats found that testosterone implants prevented a stress-induced decrease in cell proliferation [103]. However, testosterone replacement had no effect on a stress-induced decrease in cell proliferation or the number of DCX-expressing cells in the dentate gyrus among male mice [47]. A comparable experiment with rats found that testosterone implants had antidepressant effects in socially isolated males, and this effect was not associated with a change in cell proliferation in the dentate gyrus [95]. These results suggest that physiological levels of testosterone provide antidepressant effects, which are not necessarily mediated by changes in hippocampal neurogenesis. The ability of testosterone to protect against a stress-induced decrease neurogenesis seems to involve sustaining basal cell survival levels and possibly basal cell proliferation levels as well [77].

## 8. Conclusions

Besides potentially providing a better understanding of the neural mechanisms underlying memory formation, a better understanding of how sex steroids regulate adult neurogenesis holds promise for developing treatment strategies for neurological diseases that show greater prevalence in women than men, such as Alzheimer’s disease and chronic depression [221,222]. The experiments summarized here clearly demonstrate that testosterone influences adult neurogenesis, as specifically demonstrated within the HVC of birds and within the olfactory bulbs and dentate gyrus of rodents. Most work to date has been conducted using rats and mice to test the effects of a wide range of testosterone manipulations upon adult neurogenesis in the dentate gyrus. Although there are unexplained contradictions, the general conclusion from these experiments is that testosterone enhances neurogenesis by increasing the survival of newly generated neurons, while having minimal influence on levels of cell proliferation. This enhanced survival involves an androgen-dependent pathway in males, distinct from the estrogen-dependent pathway that can increase or decrease neurogenesis in females. In vitro experiments indicate that testosterone acts as a neuroprotectant, with activation of the MAPK pathway playing a key role. Some evidence indicates that BDNF is activated by androgens, but there is currently little evidence that testosterone upregulates BDNF in the dentate gyrus through a direct pathway. Indeed, there remains uncertainty regarding how androgen receptor levels are regulated in the dentate gyrus of rodents [105], and so whether testosterone binds to receptors on new neurons in the dentate gyrus directly or influences neurogenesis through indirect neural pathways is an important question to resolve. Finally, testosterone can temper the neurogenesis impairing effects of the hormonal stress response, possibly by restoring both cell proliferation and cell survival to basal levels, though it does not seem that hippocampal neurogenesis plays an essential role in the antidepressant effects of testosterone. Future work should determine the specific stages of neural development that are influenced by testosterone and determine whether testosterone has only general neuroprotective effects in the brain or if it induces unique molecular pathways in new adult neurons.

## Figures and Tables

**Table 1:** Summary of research on the effects of castration (GDX) and sex steroid manipulations on cell survival and proliferation in the subventricular zone and olfactory bulbs of male rodents relative to relevant control groups.

Species	Histological Method	Manipulation ^1^	Proliferation ^2^	Survival (Days) ^3^	Ref
mouse	BrdU	GDX	↑		[78]
rat	BrdU BrdU BrdU BrdU	GDX GDX + T GDX + DHT GDX + E2	↓ ↑ **0** ↑		[82]
mouse	BrdU	GDX + T		↓ (28)	[84]

^1^ Experimental manipulations of animals: gonadectomy (GDX); testosterone administration (+ T); estradiol administration (+ E2); DHT administration (+ DHT); ^2^ The experimental manipulation may have increased (**↑**) decreased (**↓**) or had no effect (**0**) on cell proliferation and/or cell survival. ^3^ Days refers to how old cells were at the time of assessment of cell survival, if known based on timing of BrdU injections.

**Table 2:** Summary of research on the effects of castration (GDX) and sex steroid manipulations on cell survival and proliferation in the dentate gyrus of male rodents relative to relevant control groups.

Species	Histological Method	Manipulation ^1^	Proliferation ^2^	Survival (Days) ^3^	Ref
rat	Ki67 BrdU	GDX + E2 GDX + E2	**0**	**0** (16)	[43]
rat	BrdU BrdU BrdU BrdU	GDX GDX + T GDX + DHT GDX + E2	**0**	↓ (30) ↑ (30) ↑ (30) **0** (30)	[88]
rat	Ki67 BrdU	GDX GDX	↓	↓ (24)	[89]
mouse	Ki67 DCX	GDX GDX	**0**	↓	[91]
rat	Ki67 DCX BrdU	GDX GDX GDX	**0**	**0** **0** (14)	[92]
rat	Ki67 BrdU	GDX + T GDX + T	**0**	**0** (24)	[93]
rat	BrdU	GDX + T	**0**	**0** (21)	[94]
rat	BrdU	GDX + T	**0**		[95]
mouse	Ki67 BrdU	T T	**0**	↑ (35)	[96]
vole	BrdU	GDX + T GDX + DHT GDX + E2	**0** **0** **0**		[97]
rat	Ki67	GDX + AAS	**0**		[98]
rat	BrdU DCX	GDX GDX	↓	↓	[99]
rat	BrdU BrdU BrdU BrdU	GDX + T GDX + TFM + T GDX + DHT GDX + DHT + Flu	**0** **0**	↑ (30) ↓ (30) ↑ (30) ↓ (30)	[100]
rat	BrdU	GDX GDX + T		↓ (16) **0** (16)	[101]
rat	BrdU	AAS		↓ (5)	[102]
rat	BrdU	T	**0**	**0** (26)	[103]
mouse	BrdU BrdU	T E2		**0** (28) **0** (28)	[104]
rat	Ki67 BrdU	GDX + DHT GDX + DHT	**0**	↑ (30)	[105]
mouse	Ki67 BrdU	GDX + DHT GDX + DHT	**0**	↑ (37)	[106]
mouse	BrdU DCX	Fin Fin	↓	↓	[107]
vole	BrdU	GDX + E2		↑ (16)	[108]
mouse	BrdU DCX	E2 E2	↑	**0** (21) ↑	[109]

^1^ Experimental manipulations of animals: gonadectomy (GDX); testosterone administration with or without gonadectomy (+ T); estradiol administration (+ E2); DHT administration (+ DHT); flutamide administration (+ Flu); anabolic androgenic steroids (+ AAS); testicular feminization mutation (TFM); finasteride administration (Fin). ^2^ The experimental manipulation may have increased (**↑**), decreased (**↓**), or had no effect (**0**) on cell proliferation and/or cell survival. ^3^ Days refers to how old cells were at the time of assessment of cell survival, if known based on timing of BrdU injections.
